# Protective effects of *Lactobacillus reuteri* SJ-47 strain exopolysaccharides on human skin fibroblasts damaged by UVA radiation

**DOI:** 10.1186/s40643-022-00617-0

**Published:** 2022-12-14

**Authors:** Jingsha Zhao, Hao Fu, Yongtao Zhang, Meng Li, Dongdong Wang, Dan Zhao, Jiachan Zhang, Changtao Wang

**Affiliations:** 1 Beijing Key Laboratory of Plant Resource Research and Development, College of Chemistry and Materials Engineering, Beijing Technology and Business University, Beijing, People’s Republic of China; 2 Institute of Cosmetic Regulatory Science, Beijing Technology and Business University, Beijing, People’s Republic of China

**Keywords:** *Lactobacillus reuteri* SJ-47 strain exopolysaccharides, Skin photoaging, Oxidative stress

## Abstract

Ultraviolet rays in sunlight can cause skin damage and premature aging. This study demonstrates that *Lactobacillus reuteri* SJ-47 strain exopolysaccharides (EPS) protect human skin fibroblasts (HSF) under UVA radiation. During the course of the experiments, we investigate the oxidative stress protection and antiaging effects of exopolysaccharides on HSF at the biochemical, cellular, and molecular levels. The results show that EPS can increase the antioxidant capacity of cells, decrease the amount of reactive-oxygen species (ROS) and malondialdehyde (MDA), while improve the expression of antioxidant enzymes. At the same time, EPS can increase collagen content, which can effectively regulate the expression of genes in the senescence and apoptosis pathways, and delay skin photoaging caused by UVA irradiation.

**Graphical Abstract:**

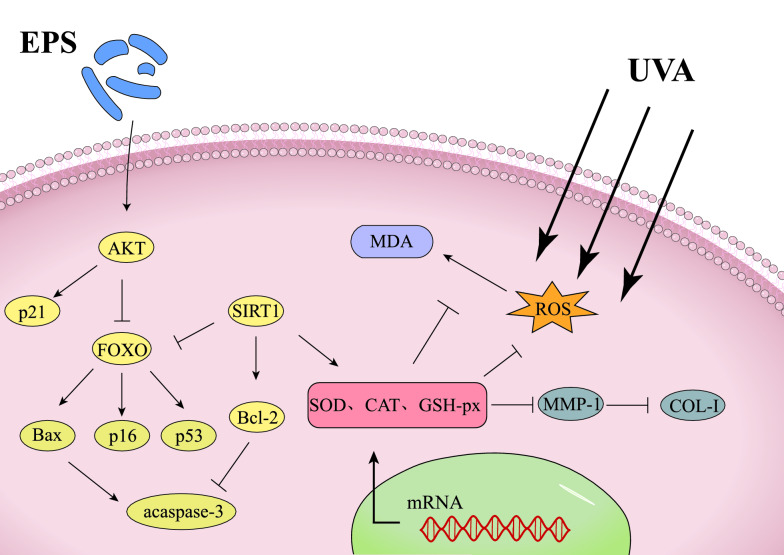

**Supplementary Information:**

The online version contains supplementary material available at 10.1186/s40643-022-00617-0.

## Introduction

The skin is the main organ that wraps the surface of the human body and protects it from external harm and damage (Chen et al. 2019). Skin aging is a progressive change in the physiology and appearance of the skin caused by the accumulation of endogenous and exogenous factors (Jenkins 2002). As the skin ages, its protective barrier is damaged. The natural aging of the skin is a gradual process, but exogenous factors can accelerate it, among which ultraviolet rays from the sun are an important external factor that triggers premature skin aging (Florence et al. 2012). Ultraviolet light can be divided into three categories according to wavelength: UVA, UVB, and UVC (Florence et al. 2012). UVA has a strong penetrating ability. It triggers endogenous photosensitization due to its penetration into the dermis of the skin and then mediates oxidative stress in skin cells, ultimately producing the appearance of skin photoaging and photoinjury ( Thomas et al. 2012). Photoaging can also be seen as external skin aging and includes sagging skin, increased wrinkles, dryness, and patchy/mottled pigmentation, and can also cause skin cancer in severe cases (You-Cheng et al. 2019). The superposition of reactive oxygen species (ROS) is one of the core mechanisms of skin aging, which accumulate in large amounts under UVA irradiation, causing oxidative stress that can lead to lipid, protein, nucleic acid and organelle damage (Yanpei et al. 2020). Ultraviolet irradiation induces the breakdown of the skin’s connective tissue by activating matrix metalloproteinases (MMPs), and the synthesis of collagen interacts with the inhibition of MMPs, both of which are closely related to skin aging and can lead to increased wrinkles and decreased elasticity (Kim et al. 2012).

Among all probiotics, *Lactobacillus* is the best producer of exopolysaccharides, and the *Lactobacillus* family is recognized as a safe organism (Kšonžeková et al. 2016; Jolly et al. 2014). *Lactobacillus reuteri* is widespread in the intestines of vertebrates and mammals, and can grow in both anaerobic and non-anaerobic environments. As a probiotic, it serves a variety of functions in humans, such as regulating the host immune system and intestinal microflora to relieve inflammation and maintain body homeostasis, secreting broad spectrum antibacterial compounds, regenerating and repairing host intestinal epithelial tissue, preventing diarrhea and colitis, improving cholesterol metabolism, reducing cardiovascular disease, promoting anti-allergic effects, maintaining oral health, relieving depression, and reducing crying in infants and young children, *Lactobacillus reuteri* has been shown to prevent and/or treat many diseases, among which infant colic is one of the main treatment diseases of *Lactobacillus reuteri*. Infant colic can cause crying behavior in infants, and the clinical efficacy of *Lactobacillus Reuteri* in human breast milk has been shown to reduce crying time (Qinghui et al. 2018).

Numerous studies have proven that polysaccharides have antioxidant and anti-aging effects on the skin. This is because they can scavenge free radicals, improve the expression of antioxidant enzymes and inhibit the content of lipid peroxides, thereby delaying skin aging caused by ultraviolet radiation (Qianru et al. 2021). At present, research on *Lactobacillus reuteri* EPS is focused on their development and application in food, and antibacterial and anti-inflammatory effects on epithelial cells (Shanmugam et al. 2020; Ispsirli et al. 2019; Butler et al. 2020), and there is no experiment to study the *Lactobacillus reuteri* EPS protection of cells damaged by UVA irradiation. Therefore, this study focuses on cellular oxidative stress induced by UVA irradiation to explore whether *Lactobacillus reuteri* EPS can protect human skin fibroblasts (HSF) under UVA radiation.

We isolated a strain of *Lactobacillus reuterii* from yogurt in the laboratory and named this strain SJ-47 and its EPS showed strong antioxidant activity in vitro. We then conducted experiments on *Lactobacillus reuteri SJ-47* strain EPS at the biochemical, cellular, and molecular levels to further validate their cellular efficacy.

## Materials and methods

### Materials

HSF (Cell Resource Center, Institute of Basic Medical Sciences, Chinese Academy of Medical Sciences); *Lactobacillus reuteri SJ-47* strain (extracted from yogurt, deposit number: CGMCC No. 16416); Fetal Bovine Serum (FBS); Dulbecco`s Modified Eagle’s Medium (DMEM); trypsin–EDTA; Phosphate Buffered Saline (PBS, Solaibao Technology, Beijing, China); penicillin–streptomycin (GIBCO Life Technologies, Grand Island, NY); Cell Counting Kit-8 (CCK8); Total Antioxidant Capacity Assay Kit with ABTS Method; Reactive-Oxygen Species (ROS) Assay Kit; malondialdehyde (MDA); superoxide dismutase (SOD); catalase (CAT); Cell lysis buffer for BCA Protein Assay Kit and Total Glutathione Peroxidase (GSH-px) Assay Kit (Beyotime Biotechnology, Shanghai, China); Human Matrix Metalloproteinase-1 (MMP-1) ELISA Kit, Human Collagen-I (COL-I) ELISA Kit and Human caspase-3 ELISA Kit (CUSBIO, Wuhan, China); FITC-Annexin V and PI Apoptosis Kit; UEIris II RT-PCR System for First-Strand cDNA Synthesis; Fast Super EvaGreen^®^ qPCR Master Mix (Biorigin (Beijing, China) Inc.).

### Extraction and purification of *Lactobacillus reuteri* SJ-47 strain EPS

First, *Lactobacillus reuteri* SJ-47 strain was inoculated in MRS medium with pH 5.8–6.5, and statically incubated overnight at 37 °C and 180 rpm to obtain fermentation broth, then centrifuged (8000 rpm for 15 min at 4 °C) to collect the supernatant. Concentrating the fermentation supernatant by rotary evaporation can effectively reduce the use of anhydrous ethanol. We mixed fermentation supernatant and ethanol with a volume ratio of 4:1, and samples were pelleted at − 4 °C for approximately 14 h. The samples were dissolved with water, 2% volume of papain was added, the two were mixed thoroughly, the reaction was carried out at room temperature for 3 h and boiled for 15 min to inactivate the papain. The precipitate was collected after the second alcohol precipitation, and crude EPS were obtained after lyophilization. Finally, we obtained *Lactobacillus reuteri* SJ-47 strain EPS using DEAE-52 anion column chromatography and tracking detection using the sulfuric acid−phenol method (M DuBois, et al. 2002).

### Cellular analysis of *Lactobacillus reuteri* SJ-47 strain EPS

#### Cell culture

The HSF used for the experiments were between 5 and 20 generations. The HSF were placed in a CO_2_ incubator (Heracell™ VIOS 250i, Thermo Fisher Scientific (China) Co., Ltd.) and grown in a complete DMEM medium (containing 1% penicillin–streptomycin and 10% FBS). The medium was changed every 48 h and dissociated using 0.25% trypsin and 0.02% EDTA. All tests were carried out for 12 h after the cells were seeded at 1 × 10^4^ cells/well in 96-well microplates (or 5 × 10^5^ cells/well in 6-well microplates).

#### Cell viability

The HSF were seeded in a 96-well plate at 1 × 10^4^ cells/well and grown in a complete DMEM medium, washed with PBS after 12 h and divided into the model group, blank control group and sample group. The HSF were then treated with serum-free DMEM and EPS at different concentrations (10–1000 µg/mL). Cells in the control group and model group were added with serum-free DMEM, and six parallel lines were made for each sample. After one day of culture, HSF were washed with PBS, and the sample group and model group were induced with a UVA light box. The cells were 20 cm away from the UVA light (Spectronics Co., Ltd., USA), irradiation time of 1 h, irradiation intensity of 5mW/cm^2^, and final total energy of 18 J/cm^2^,while the control group was not subjected to UVA irradiation. Three other parallel samples of different concentrations were not irradiated. After UVA irradiation, the cells were rinsed twice with PBS and freshly cultured with serum-free DMEM. After 12 h of incubation, 10 µL of CCK-8 solution was added, followed by incubation for 2 h and assay at 450 nm with a fluorescence microplate reader.

#### Cell apoptosis assay

We carried out experiments on cells to determine the effect of different concentrations of EPS on cell apoptosis and the changes of cell apoptosis under UVA irradiation. The HSF were seeded in 6-well plates at 5 × 105 cells/well and grown in a complete DMEM medium. After 12 h of incubation, HSF were washed with PBS, and the HSF were incubated with serum-free DMEM and EPS at various concentrations (100–500 µg/mL). Cells in the control group and model group were added with serum-free DMEM. After one day of culture, HSF were washed with PBS, and the sample and model groups were stimulated with UVA with a total energy of 18 J/cm2, while the control group was not irradiated. After UVA irradiation, the cells were rinsed twice with PBS and freshly cultured with serum-free DMEM. After 12 h of incubation, treated twice with PBS, the cells were digested with trypsin and collected for subsequent experiments. To investigate the specificity of EPS to UVA irradiation, the effects of different concentrations of EPS on cell apoptosis were studied without UVA irradiation. All experiments were performed according to the instructions.

#### Determination of antioxidant capacity

Intracellular antioxidant levels can be measured in three parts. First, the antioxidant capacity of the cells was evaluated by assessing the intracellular free radical scavenging rate and ROS content. Second, the lipid oxidation level of cells with different EPS concentrations was judged by measuring the content of MDA in the cells. Finally, the protective effects of different concentrations of EPS on antioxidant enzymes (CAT, GSH-px and SOD) under UVA irradiation were determined. The HSF were seeded in 6-well plates at 5 × 10^5^ cells/well and grown in a complete DMEM medium. After 12 h of incubation, HSF were washed with PBS, and the HSF were incubated with serum-free DMEM and EPS at various concentrations (100–500 µg/mL). Cells in the control group and model group were added with serum-free DMEM. After one day of culture, HSF were washed with PBS, and the sample and model groups were stimulated with UVA with a total energy of 18 J/cm^2^, while the control group was not irradiated. After UVA irradiation, the cells were rinsed twice with PBS and freshly cultured with serum-free DMEM. After 12 h of incubation, treated twice with PBS and collected for detection. In order to investigate the specificity of EPS to UVA irradiation, the effects of different concentrations of EPS on the antioxidant capacity of cells and the changes in the activity of intracellular antioxidant enzymes were studied without UVA irradiation. All experiments were performed according to the instructions.

#### ELISA

The contents of COL-I, MMP-1, and caspase-3 in the cells were determined by enzyme-linked immunosorbent assay (ELISA), and then the senescence and apoptosis of the cells were analyzed. The HSF were seeded into 6-well plates at 5 × 10^5^ cells/well and grown in a complete DMEM medium. 12 h after inoculation, the HSF were incubated with serum-free DMEM and EPS of different concentrations (100–500 µg/mL), and cells in the control group and model group were added with serum-free DMEM for 24 h. HSF were washed with PBS and the sample and model groups were stimulated with UVA with a total energy of 18 J/cm^2^, while the control group was not irradiated. After UVA irradiation, the cells were rinsed twice with PBS and freshly cultured with serum-free DMEM. After 12 h of incubation, treated twice with PBS and collected for detection. To study the specificity of EPS to UVA irradiation, the contents of collagen, matrix metalloproteinase and caspase-3 were simultaneously determined by different concentrations of EPS without UVA irradiation. All experiments were performed according to the instructions.

### RT-qPCR

RT-QPCR was used to measure the expression of antioxidant enzyme genes CAT, SOD and GSH-px, anti-aging genes COL-I and MMP-1 and pro-apoptotic protease caspase-3. The expression of genes related to the cell senescence and apoptosis pathways (Bax, Bcl-2, SIRT1, p16, p53, AKT, p21, FOXO) was detected by adding EPS and irradiating with UVA. The HSF were seeded in 6-well plates at 5 × 10^5^ cells/well and grown in a complete DMEM medium. After 12 h of incubation, the HSF were incubated with serum-free DMEM and EPS (100, 250, 500 µg/mL) of different concentrations, and cells in the control group and model group were added with serum-free DMEM. After one day of culture, HSF were washed with PBS and the sample and model groups were stimulated with UVA with a total energy of 18 J/cm^2^, while the control group was not irradiated. After UVA irradiation, the cells were rinsed twice with PBS and freshly cultured with serum-free DMEM. After 12 h of incubation, treated twice with PBS and collected for subsequent testing. At the same time, we studied the effect of different concentrations of EPS on the genes in the cells without UVA. The total RNA was extracted using Trizol and the final cDNA synthesis and qPCR operations were performed using an UEIris II RT-PCR System for First-Strand cDNA Synthesis (BN12028) Inc.) and Fast Super EvaGreen^®^ qPCR Master Mix (BN12008), respectively. The primer sequence information is shown in Table 1:

**Table 1:** List of Primer Sequences

Primer name	
β-actin	F: 5′-CTGAAGCCCCACTCAATCCA -3′ R: 5′-GCCAAGTCAAGACGGAGGAT -3′
CAT	F: 5′-CCTTCGACCCAAGCAA-3′ R: 5′-CGATGGCGGTGAGTGT-3′
SOD	F: 5′-TGGAGATAATACAGCAGGCT-3′ R: 5′-AGTCACATTGCCCAAGTCTC-3′
GSH-px	F: 5′-AGAAGTGCGAGGTGAACGGT-3′ R: 5′-CCCACCAGGAACTTCTCAAA-3′
COL-I	F: 5′- CCTGGTCCTCCTGGTAGT-3′ R: 5′-TCCCTTCTCTCCTGGTTG-3′
MMP-1	F: 5′-TTGAGAAGCCTTCCAACTCTG-3′ R: 5′-CCGCAACACGATGTAAGTTGTA-3′
Caspase-3	F: 5′-TGCTATTGTGAGGCGGTTGT-3′ R: 5′-TCACGGCCTGGGATTTCAAG-3′
Bax	F: 5′-CGGAATTCATGGACGGGTCCGGGGAG-3′ R: 5′-CCGCTCGAGTCAGCCCATCTTCTTCCAG-3′
Bcl-2	F: 5′-ATGTGTGTGGAGAGCGTCAACC-3′ R: 5′-CAGAGACAGCCAGGAGAAATCAA-3′
SIRT1	F: 5′-CAAGGGATGGTATTTATGCTCG-3′ R: 5′-CAAGGCTATGAATTTGTGACAGAG-3′
p16	F: 5′-AGCCTTCGGCTGACTGGCTGG-3′ R: 5′-CTGCCCATCATGACCTGG-3′
p53	F: 5′-CTTTGAGGTGCGTGTTTGTGC-3′ R: 5′-GGTTTCTTCTTTGGCTGGGGA-3′
AKT	F: 5′-GCCGCTGCTTCTTTATCC-3′ R: 5′-GCCATTCTCCACTCCACC-3′
p21	F: 5′-GAGGCCGGGATGAGTTGGGAGGAG-3′ R: 5′-CAGCCGGCGTTTGGAGTGGTAGAA-3′
FOXO	F: 5′-TGAGGGTTAGTGAGCAGGTTAC-3′ R: 5′-AGGGAGTTGGTGAAAGACATC-3′

### Statistical analysis

Three separate experiments with three technical replicates were performed for each sample. The results were expressed as mean ± standard deviation (mean ± SD) by one-way analysis of variance (ANOVA) to determine which factors were significantly different. GraphPad Prism9 was used for statistical analysis. *P* < 0.05 was considered statistically significant.

## Results

### Effects of EPS on UVA-irradiated HSF cell viability

**Fig. 1:**
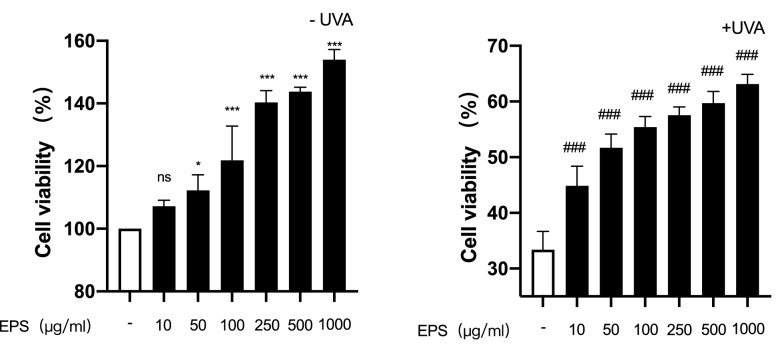
Effects of different concentrations of EPS on HSF survival rate under normal conditions and after UVA irradiation. *p  < 0.05, **p < 0.01, ***p < 0.001 compared to untreated control group; #*p* < 0.05, ##*p* < 0.01, ###*p* < 0.001, compared to model group

In this study, a CCK8 assay was used to determine the cytotoxicity of EPS and the protective effects of EPS addition on HSF under UVA irradiation. As shown in Fig. 1, EPS has no toxic effects on cells at concentrations between 10 and 1000 µg/mL. At the same time, the cell activity rate increased significantly with the increase in EPS concentration, which suggests that EPS has a proliferation effect on cells. Meanwhile, cells were obviously damaged after UVA radiation and cell viability increased with the increase in EPS concentration. This phenomenon further confirmed that EPS has certain protective effects and helps to restore damage caused by UVA to HSF cells. In view of the protective effect of EPS on cells after UVA irradiation, EPS concentrations of 100, 250, and 500 µg/mL were used for subsequent tests.

### Effects of different concentrations of EPS on cell apoptosis

**Fig. 2:**
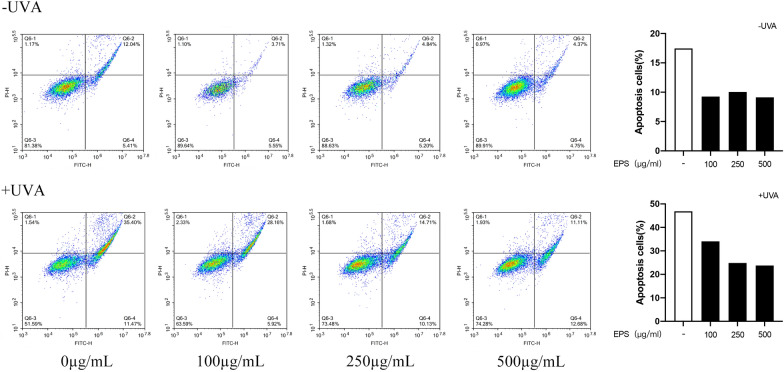
Effects of different concentrations of EPS on HSF apoptosis under normal conditions and after UVA irradiation

FITC-Annexin V and PI Apoptosis staining was used to stain HSF. The experimental results were shown in Fig. 2, EPS showed obvious inhibition of HSF apoptosis under normal conditions. The apoptosis rate was about 10% when the EPS concentration was 100, 250, and 500 µg/mL. After irradiation with UVA, the apoptosis rate of the model group was significantly increased, reaching nearly 50%. After pretreatment with EPS, the apoptosis rate of the cells decreased. It can be seen that when EPS concentration was 500 µg/mL, the apoptosis rate of the cells decreased to 23.8%, which played an obvious inhibitory effect on the apoptosis of the cells.

### Effects of EPS on ABTS, ROS, and MDA in UVA-irradiated HSF cells

**Fig. 3:**
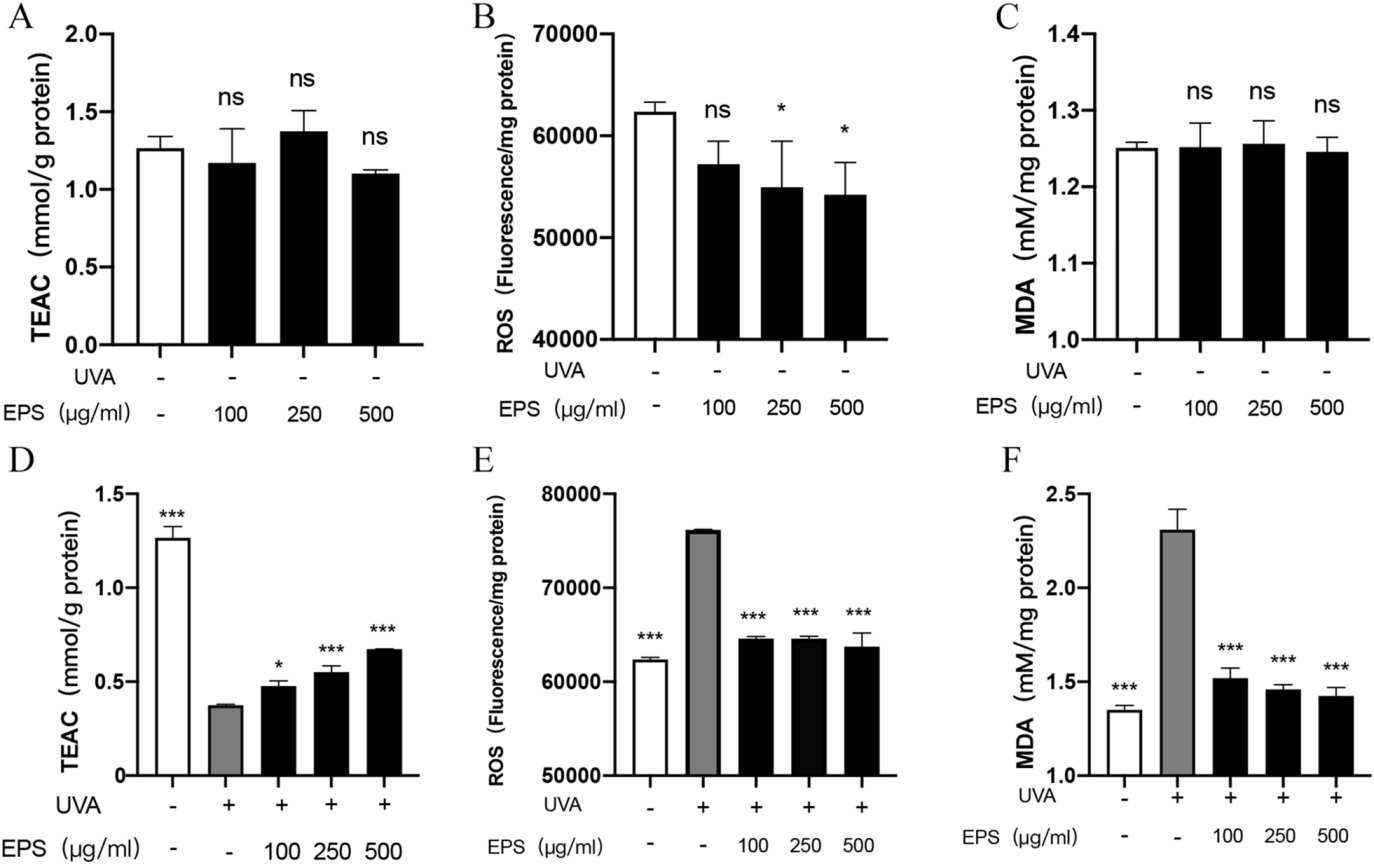
Effects of different concentrations of EPS on total antioxidant capacity (ABTS), ROS scavenging capacity and MDA content: **A** Determination of Trolox Equivalent Antioxidant Capacity (TEAC) under normal conditions; **B** Fluorescence intensity of HSF under normal conditions; **C** Malondialdehyde content of HSF under normal conditions; **D** Determination of Trolox Equivalent Antioxidant Capacity (TEAC) under UVA irradiation; **E** Fluorescence intensity of HSF under UVA irradiation; **F** Malondialdehyde content of HSF under UVA irradiation. *p < 0.05, **p < 0.01, ***p < 0.001 compared to model group

EPS measured the antioxidant capacity, ROS content and lipid oxidation of the cells under normal conditions, and it was found that the total antioxidant capacity (3A) and lipid oxidation level of the sample group and the control group were not significantly different (3C). EPS significantly reduced the content of reactive oxygen species (3B) at concentrations of 250 µg/mL and 500 µg/mL. EPS can reduce the oxidative stress of cells under UVA irradiation and thus alleviate oxidative damage. First, we detected the antioxidant capacity of the HSF cells (Fig. 3D), which decreased significantly after UVA irradiation. With the increase in EPS concentration, the antioxidant capacity of the cells increased, presenting an apparent dose-dependent relationship. Afterwards, the ROS content in the HSF cells was detected (Fig. 3E), and it was found that ROS increased significantly after UVA irradiation. Treating HSF with EPS can reduce the content of ROS produced under UVA irradiation, and the ROS content can be basically restored to the same level as that of the control group. Cellular oxidative stress was accompanied by lipid oxidation, and the degree of lipid oxidation was judged by measuring the MDA content (Fig. 3F). The MDA content was significantly decreased in HSF treated with EPS.

### Effects of EPS on CAT, SOD, and GSH-px activity in UVA-irradiated HSF cells

**Fig. 4:**
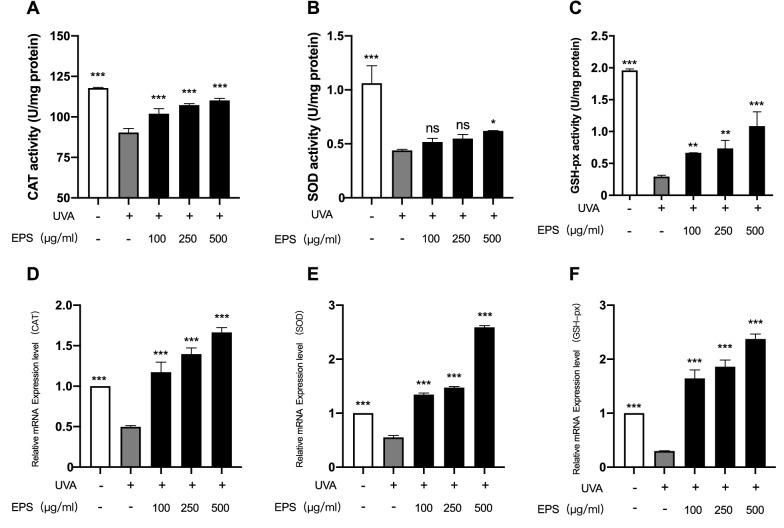
Effects of different concentrations of EPS on antioxidant enzymes CAT (**A**), SOD (**B**) and GSH-px (**C**) in HSF under UVA irradiation. Relative mRNA expression levels of CAT (**D**), SOD (**E**) and GSH-px (**F**) under UVA irradiation after treatment with different concentrations of EPS. Each mRNA was referenced to the internal control gene β-actin and expressed relative to the control group. *p < 0.05, **p < 0.01, ***p < 0.001 compared to model group

CAT, SOD, and GSH-px are common antioxidant enzymes in the antioxidant system of cells. Antioxidant enzymes protect the organelles from oxidative damage by removing free radicals and ROS produced in cells. The contents of CAT, SOD, and GSH-px were measured by EPS under normal conditions, and the relative mRNA expression levels of these three antioxidant enzymes were also detected. The experimental results showed no significant difference in the contents of antioxidant enzymes and relative mRNA expression levels between the sample group and the control group (Additional file 1). After UVA irradiation, it can be seen from Fig. 4A that the CAT content in cells decreased significantly, while EPS could weaken the trend of CAT decline in HSF cells. When combined with Fig. 4D, it can be seen that EPS could induce HSF cells to increase the expression of CAT mRNA, and the CAT in cells has a significant upward trend when the concentration of EPS increased. It can be seen from Figs. 4B and 4E that the SOD content in cells decreased significantly after UVA irradiation. When the EPS concentration was 500 µg/mL, the relative expression of SOD mRNA in the cells was significantly increased, and the SOD content in the cells was also increased. It can be seen from Fig. 4C that the GSH-px content in cells was significantly reduced after UVA irradiation, and when the EPS concentration was 500 µg/mL, the GSH-px content was significantly increased (*P* < 0.001). As can be seen from Fig. 4F, the GSH-px mRNA expression level was significantly increased under different concentrations of EPS. In conclusion, EPS can promote the increase of the mRNA relative expression level of antioxidant enzymes in HSF after UVA irradiation and repair oxidative stress damage by increasing the activity of CAT, SOD, and GSH-px.

### Effects of EPS on UVA-irradiated COL-I, MMP-1, and caspase-3 contents

**Fig. 5:**
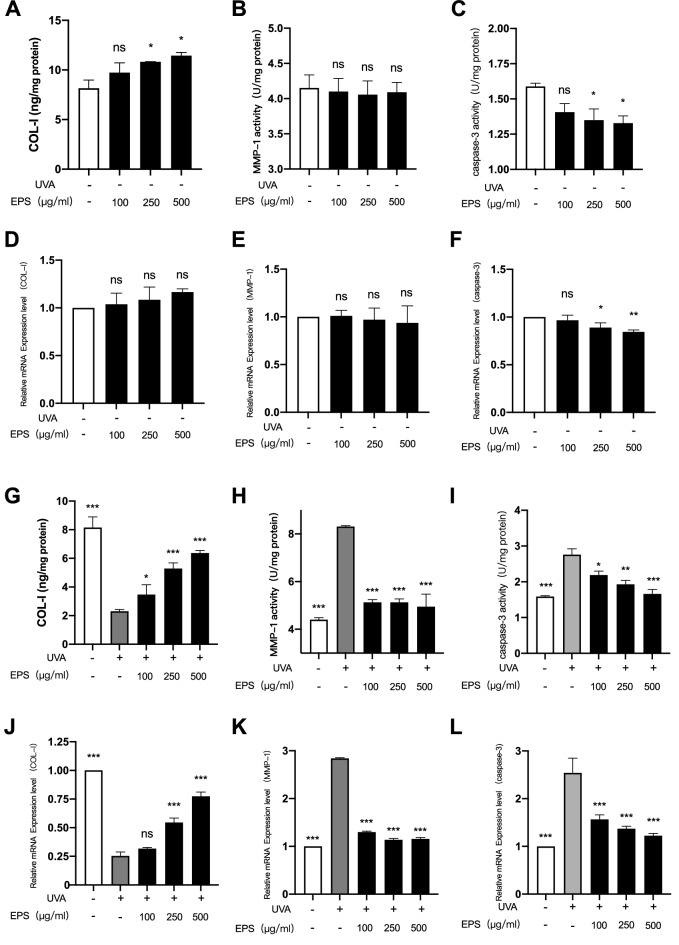
Effects of different concentrations of EPS on COL-I (**A**), MMP-1 (**B**) and caspase-3 (**C**) in HSF under normal conditions. Relative expression levels of COL-I (**D**), MMP-1 (**E**) and caspase-3 (**F**) mRNA in HSF under normal conditions with different concentrations of EPS. Effects of different concentrations of EPS on COL-I (**G**), MMP-1 (**H**) and caspase-3 (**I**) in HSF under UVA irradiation. Relative expression levels of COL-I (**J**), MMP-1 (**K**) and caspase-3 (**L**) mRNA in HSF under UVA irradiation with different concentrations of EPS.Each mRNA was referenced to the internal control gene β-actin and expressed relative to the control group. *p < 0.05, **p < 0.01, ***p < 0.001 compared to model group

A major factor in skin aging is the loss of collagen and the degradation of the extracellular matrix, while the latter can affect the synthesis of collagen. In this study, COL-I and MMP-1 contents and mRNA relative expression levels were measured to evaluate whether EPS had a certain delaying effect on cell senescence. Caspase-3 is one of the key enzymes in the process of cell apoptosis. After aging, cells will go on the road of apoptosis. We determined whether EPS has an inhibitory effect on cell apoptosis by measuring caspase-3. We measured the contents of COL-I, MMP-1 and caspas-3 of EPS in HSF under normal conditions, and found that the contents of COL-I (5A) and MMP-1 (5B) did not change significantly, and the relative mRNA expression levels (5D and 5E) were not significantly different. However, the activity of caspase-3 (5C) gradually decreased with the increase of concentration, and its mRNA relative expression level (5F) was lower than that of control group. It can be seen from Fig. 5G, the content of COL-I in HSF were significantly reduced. Under UVA irradiation after EPS pretreatment, the content of COL-I in HSF were significantly increased. It can be seen from Fig. 5J, EPS obviously increased the mRNA expression level of COL-I in UVA-induced cells. This indicates that EPS can promote the generation of collagen after oxidative stress. As shown in Fig. 5H that the content of cell matrix metalloenzyme increased significantly after UVA irradiation, indicating that oxidative stress can promote the accelerated degradation of the extracellular matrix, while EPS can inhibit the generation of MMP-1. When combined with Fig. 5K, it can be seen that when the concentration of EPS was 500 µg/mL, MMP-1 mRNA expression was significantly decreased (P < 0.001). As shown in Fig. 5I, after UVA irradiation, the content of caspase-3 in cells significantly increased, while the content of caspase-3 in cells pretreated with EPS showed a downward trend. When combined with Fig. 5L, it can be found that the relative expression level of caspase-3 mRNA in cells decreased significantly after EPS pretreatment. These results indicated that EPS could inhibit the expression of caspase-3 to some extent. In conclusion, EPS can improve premature aging caused by UVA irradiation, delay skin aging, and inhibit cell apoptosis.

### Effects of EPS on UVA-irradiated cell senescence and apoptosis pathways

**Fig. 6:**
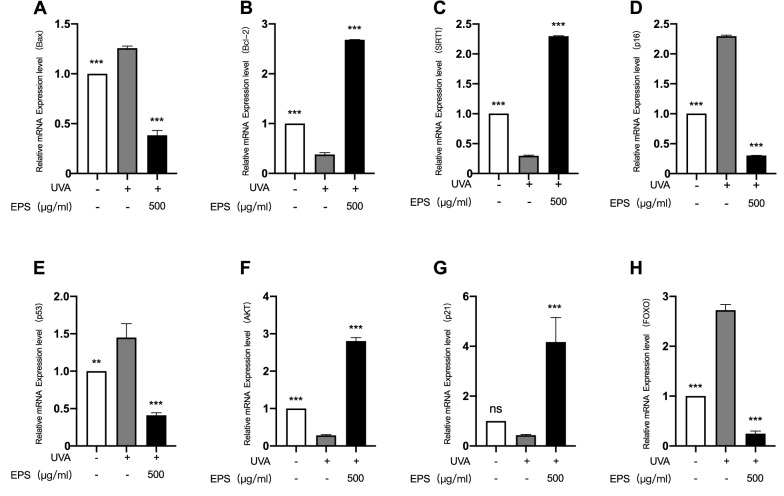
Effects of different concentrations of EPS on the expression of  **A** Bax, **B** Bcl-2, **C** SIRT1, **D** p16, **E** p53, **F** AKT, **G** p21 and **H** FOXO in the HSF senescence and apoptosis pathways induced by UVA. Each mRNA was referenced to the internal control gene β-actin and expressed relative to the control group. *p < 0.05, **p < 0.01, ***p < 0.001 compared to model group

Based on the above experimental data, we finally chose to detect the mRNA relative expression levels of the HSF senescence and apoptosis pathways under normal conditions and after UVA irradiation when the EPS concentration was 500 µg/mL. Under normal conditions, EPS has no obvious effect on the relative mRNA expression levels of HSF senescence and apoptosis pathways (Additional file 2). Under UVA irradiation, it can be seen from Fig. 6A that the relative expression level of Bax mRNA in HSF cells was up-regulated, while it was significantly down-regulated and obviously lower than that of the control group after EPS pretreatment. It can be seen from Fig. 6B, the relative expression level of Bcl-2 mRNA in HSF were down-regulated after UVA irradiation, while it was significantly up-regulated after EPS pretreatment to 2.7 times that of the control group. As can be seen from Fig. 6C that the relative expression level of SIRT1 mRNA in HSF were down-regulated after UVA irradiation, while it was significantly up-regulated and obviously higher than that of the control group after EPS pretreatment. It can be seen from Fig. 5D, the relative expression level of p16 mRNA in HSF cells was obviously up-regulated after UVA irradiation, while it was significantly down-regulated after EPS pretreatment and obviously lower than that of the control group. It can be seen from Fig. 6E, the relative expression level of p53 mRNA in HSF cells was up-regulated after UVA irradiation, while it was down-regulated after EPS pretreatment to 0.4 times that of the control group. Figure 6F shows that the relative expression level of AKT mRNA in HSF cells was down-regulated after UVA irradiation, while it was significantly up-regulated after EPS pretreatment. As can be seen from Fig. 6G, the relative expression level of p21 mRNA in HSF cells was down-regulated after UVA irradiation, while it was significantly up-regulated after EPS pretreatment to four times that of the control group. As can be seen from Fig. 6H, the relative expression level of FOXO mRNA in HSF cells was significantly up-regulated after UVA irradiation, while it was significantly down-regulated after EPS treatment. In conclusion, when the EPS concentration was 500 µg/mL, the expression of key genes in the UVA-induced HSF cell senescence and apoptosis pathways was regulated to repair the oxidative stress damage to cells.

Transcription factor forkhead box protein O (FOXO) is a downstream effector of AKT, and the expression of FOXO target genes regulates cell growth, apoptosis, and senescence (Hou et al. 2016). FOXO is phosphorylated and degraded by AKT (Wolfgang et al. 2009), and the inhibition of AKT phosphorylation in the presence of apoptotic factors facilitates the translocation of dephosphorylated FOXO into the nucleus and triggers the expression of apoptosis-related genes (Bax, p16, p21, and p53, etc.), which leads to cell growth cycle arrest and apoptosis (Orabi Sahar Hassan et al 2020; Zhixue et al. 2019; Jesel et al. 2019; Jose and Kurup 2017). Among them, the p21 gene can resist apoptosis, and at the same time it inhibits proliferation and promotes apoptosis. On the one hand, p21 can cause cell cycle arrest, giving cells extra time to repair damage; on the other hand, p21 upregulation promotes apoptosis, so it has an antagonistic duality (Jose and Kurup 2017). However, SIRT1 can inhibit the expression of apoptotic factors through the deacetylation of p53 and FOXO, and promote the expression of genes related to cell damage repair and growth (SOD, CAT, and Bcl-2, etc.) (XiaoHu et al. 2021; Lulu et al. 2019).

According to other studies, excessive UV radiation promotes phenomena, such as apoptosis and stress damage, which lead to skin aging. To investigate and analyze the value of *Lactobacillus reuteri* SJ-47 strain EPS in skin protection, a UVA-induced HSF model was established to investigate the oxidative stress protective and anti-aging effects of EPS on HSF at the biochemical, cellular, and molecular levels, and our findings are consistent with this conclusion. The downregulation of AKT and SIRT1 expression and upregulation of FOXO expression in HSF under UVA stimulation at 18 J/cm2 indicated the activation of the FOXO apoptotic pathway. This was evidenced by the marked upregulation of the expression levels of downstream pro-apoptotic factors Bax, p16 and p53. Fortunately, the level of the activated FOXO apoptotic pathway was extremely significantly decreased in EPS-treated HSF when they were stimulated by UVA again. In particular, the expression levels of AKT, SIRT1, and anti-apoptotic factor Bcl-2 were extremely significantly up-regulated, which largely inhibited the nuclear displacement and acetylation levels of FOXO, thereby reducing the expression of apoptotic factors, while the upregulation of p21 causes cell cycle arrest to repair cell damage, and ultimately promotes cell growth and oxidative stress repair, as evidenced by the increased activity and expression levels of cellular antioxidant enzymes and increased cell survival rate. For example, under UVA stimulation, the survival rate of HSF incubated with EPS increased to 64% from 32% without incubation, and its HSF apoptosis rate decreased from 47 to 24% without incubation, while the intracellular antioxidant enzymes (SOD, CAT, and GSH-px) showed a significant increase in enzyme activity and expression level. Under the incubation of 500 µg/mL EPS, the antioxidant capacity of cells was significantly increased, and the contents of ROS and MDA were obviously decreased. In addition, we found that the expression of MMP-1 in HSF after EPS incubation was inhibited and the corresponding enzyme activity was significantly reduced, while the expression level of Type I procollagen and extracellular COL-I content were significantly up-regulated, all of which resulted in the optimal effects of EPS at 500 µg/mL, effectively alleviating skin aging caused by the degradation of the extracellular matrix due to UVA irradiation.

In conclusion, *Lactobacillus reuteri SJ-47* strain EPS have strong photoprotective effects on UVA-irradiated HSF, giving them great significance in the regulation of the cell senescence and apoptosis pathways, and great potential value in cosmetic applications. However, further experiments are required to analyze the single components of *Lactobacillus reuteri SJ-47* strain EPS and explore their physical and chemical properties. At the same time, in addition to the antioxidant effects of *Lactobacillus reuteri SJ-47* strain EPS, we can also study their other effects on cells and further determine whether EPS can become a functional raw material for cosmetics (Additional file 1 and 2).

### Supplementary Information

**Additional file 1.** Effects of different concentrations of EPS on antioxidant enzymes CAT (A), SOD (B) and GSH-px (C) in HSF under normal conditions. Relative mRNA expression levels of CAT (D), SOD (E) and GSH-px (F) under normal conditions with different concentrations of EPS. Each mRNA was referenced to the internal control gene β-actin and expressed relative to the control group.

**Additional file 2.** Effects of different concentrations of EPS on the expression of (A) Bax, (B) Bcl-2, (C) SIRT1, (D) p16, (E) p53, (F) AKT, (G) p21 and (H) FOXO in the HSF senescence and apoptosis pathways under normal conditions. Each mRNA was referenced to the internal control gene β-actin and expressed relative to the control group.

## Data Availability

The datasets used during the current study are available from the corresponding author on reasonable request.

## Abbreviations

EPS: *Lactobacillus reuteri* SJ-47 strain exopolysaccharides
HSF: Human skin fibroblasts
ROS: Reactive-oxygen species
MDA: Malondialdehyde
MMPs: Matrix metalloproteinases
FBS: Fetal bovine serum
DMEM: Dulbecco: s modified Eagle’s medium
PBS: Phosphate buffered saline
CCK8: Cell counting kit-8
SOD: Superoxide dismutase
CAT: Catalase
GSH-px: Glutathione peroxidase
MMP-1: Matrix metalloproteinase-1
COL-I: Human collagen-I
ELISA: Enzyme-linked immunosorbent assay
FOXO: Transcription factor forkhead box protein O
