# Supplementary figures and images for: Protective effects of Lactobacillus reuteri SJ-47 strain exopolysaccharides on human skin fibroblasts damaged by UVA radiation
Source: Bioresour Bioprocess. 2022 Dec 14;9(1):127. doi: 10.1186/s40643-022-00617-0 (PMC10992028; doi:10.1186/s40643-022-00617-0)

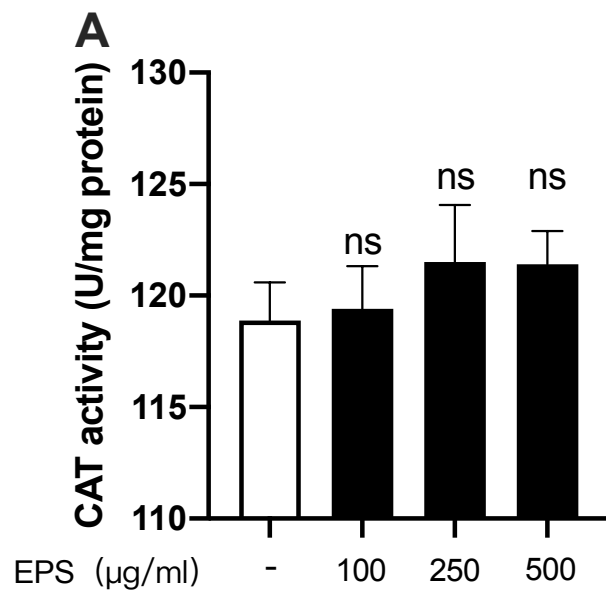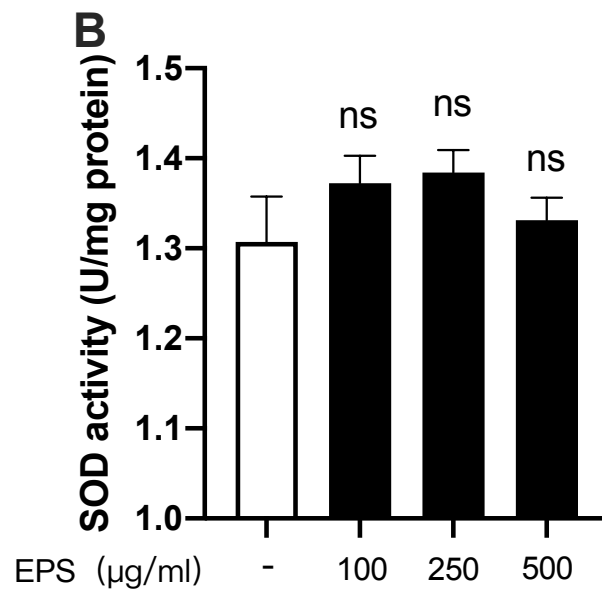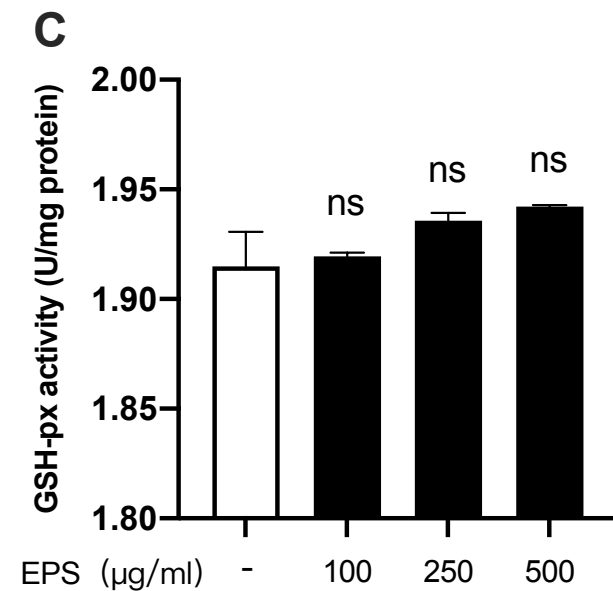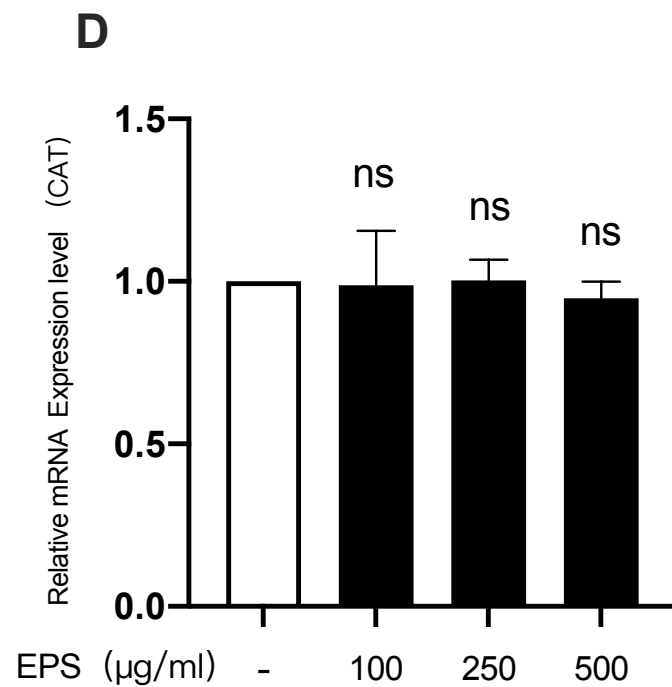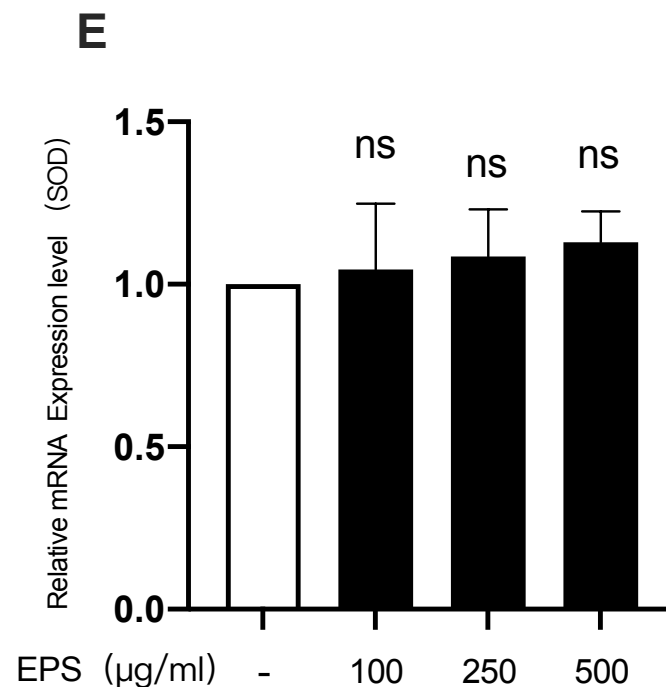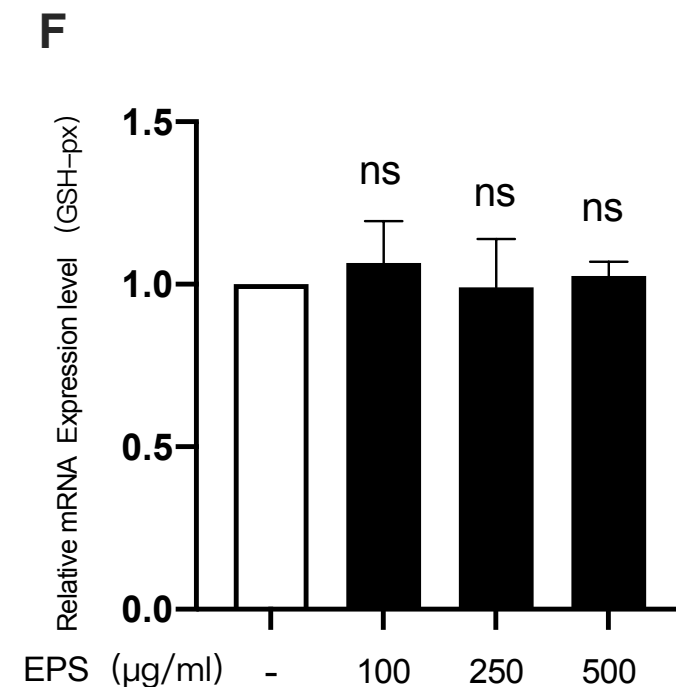

Supplement: Supplementary file 1 — Additional file 1. Effects of different concentrations of EPS on antioxidant enzymes CAT (A), SOD (B) and GSH-px (C) in HSF under normal conditions. Relative mRNA expression levels of CAT (D), SOD (E) and GSH-px (F) under normal conditions with different concentrations of EPS. Each mRNA was referenced to the internal control gene β-actin and expressed relative to the control group. [file 40643_2022_617_MOESM1_ESM.pdf]

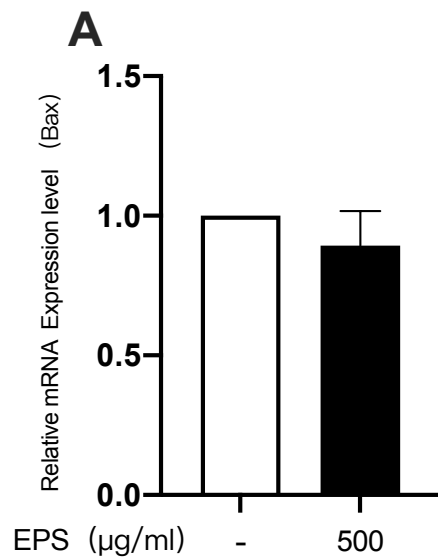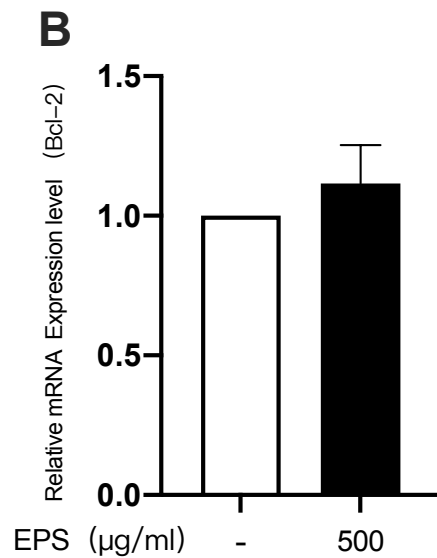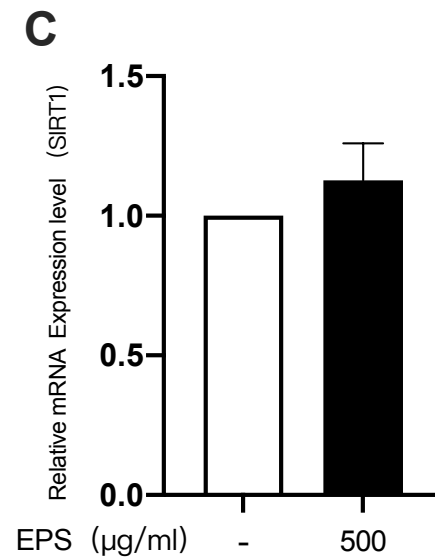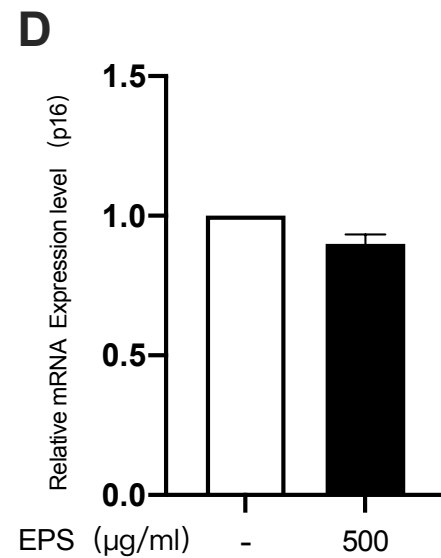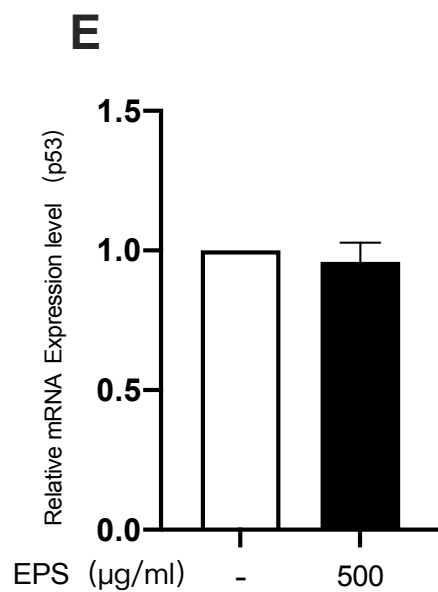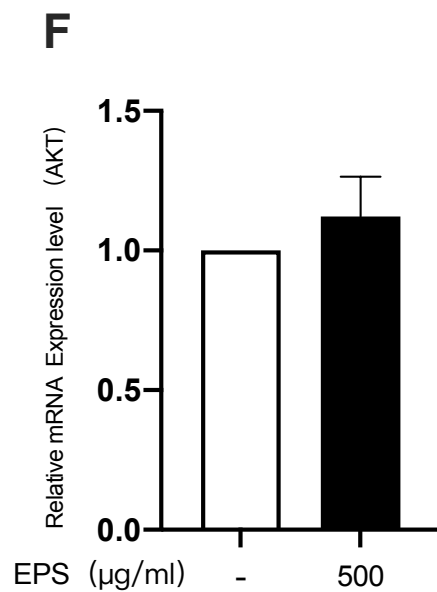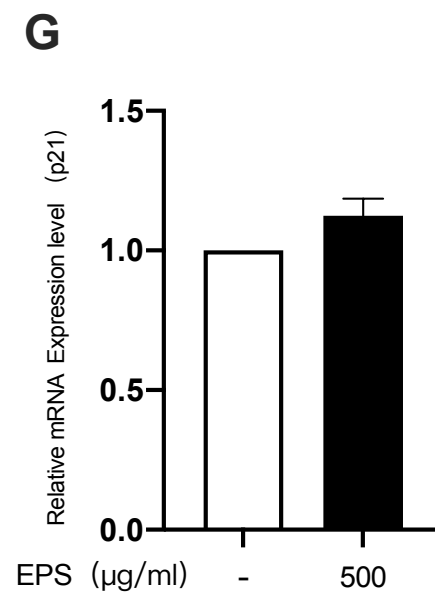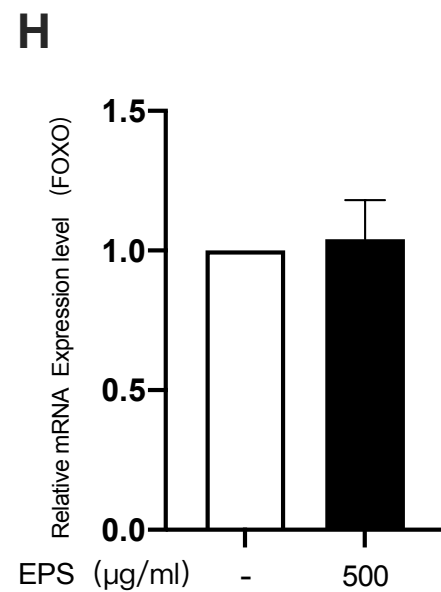

Supplement: Supplementary file 2 — Additional file 2. Effects of different concentrations of EPS on the expression of (A) Bax, (B) Bcl-2, (C) SIRT1, (D) p16, (E) p53, (F) AKT, (G) p21 and (H) FOXO in the HSF senescence and apoptosis pathways under normal conditions. Each mRNA was referenced to the internal control gene β-actin and expressed relative to the control group. [file 40643_2022_617_MOESM2_ESM.pdf]
